# Considerations for therapeutic intervention models of pulmonary fibrosis

**DOI:** 10.1186/1476-9255-10-S1-P39

**Published:** 2013-08-14

**Authors:** JA Wilder, M Doyle-Eisele, KM Gott, Y Shi, R Guilmette, A Gigliotti, J McDonald, IO Rosas

**Affiliations:** 1 Lovelace Respiratory Research Institute and Harvard University, Brigham and Women’s Hospital, USA

## 

Our laboratory has evaluated therapeutic strategies for pulmonary fibrosis in rodents, humans and recently non-human primates. Clinically, our studies have evaluated biomarkers of susceptibility to fibrosis that may lead to new innovations on treatment strategies, prevention, and biomarkers. It is anticipated that these biomarkers may also be applied in animal models of disease. Modeling idiopathic pulmonary fibrosis in animals is complicated by the complexity of the disease, and often limited by limitations in the model. Bleomycin induced fibrosis has been extensively characterized and is the workhorse of the pharmacology community. The utility of this model is limited by its ability to resolve and its relatively artificial mode of action (although there are many commonalities that have been confirmed in humans). We have evaluated permutations of the model to optimize the homogeneity and stability of the lung injury from Bleomycin treatment, including delivering it by inhalation in both rodents and recently non-human primates (NHP). The model can be utilized in a number of ways to evaluate therapeutic intervention. A limitation is that many compounds which have been successful at mitigating bleomycin induced injury in the model have not been successful at mitigating human fibrosis. As a result, we have enhanced our models to permit a two stage evaluation that uses radiation induced pulmonary fibrosis subsequent to bleomycin. Mice are irradiated once and develop fibrosis between 16-24 weeks afterwards. The advantage of this approach is that the mechanism of fibrotic progression has some complements to bleomycin but also some important contrasts. A disadvantage is the time frame of the model, which makes it of limited utility for relatively rapid screening of molecules. The important lesson learned from our combined preclinical and clinical model evaluations is that it is important to continue to utilize clinical and translational techniques (including *in vitro*) to determine the mode of action in addition to the pharmacology. Because of the large number of false positives that have been observed in the bleomycin fibrosis model, it is important to complement it with alternative approaches such as radiation and examination in multiple species (e.g., NHP). Further, the nonclinical data must offer informative mechanistic information both to enhance the biomarkers available in the clinic and to ensure that the mode of action is feasible in humans. We will present information on several of these models, utilizing case studies of several recent published therapeutics and therapeutic pathways.

